# Three‐dimensional bioprinting of stem cell‐derived central nervous system cells enables astrocyte growth, vasculogenesis, and enhances neural differentiation/function

**DOI:** 10.1002/bit.28470

**Published:** 2023-07-03

**Authors:** Michael A. Sullivan, Samuel Lane, Alexander Volkerling, Martin Engel, Eryn L. Werry, Michael Kassiou

**Affiliations:** ^1^ School of Medical Sciences, The Faculty of Medicine and Health The University of Sydney Sydney New South Wales Australia; ^2^ School of Chemistry, The Faculty of Science The University of Sydney Sydney New South Wales Australia; ^3^ Inventia Life Science Operations Pty Ltd. Alexandria New South Wales Australia; ^4^ Central Clinical School, Faculty of Medicine and Health The University of Sydney Sydney New South Wales Australia

**Keywords:** 3D, bioprinting, CNS, hydrogel, iPSC, poly(ethylene glycol)

## Abstract

Current research tools for preclinical drug development such as rodent models and two‐dimensional immortalized monocultures have failed to serve as effective translational models for human central nervous system (CNS) disorders. Recent advancements in the development of induced pluripotent stem cells (iPSCs) and three‐dimensional (3D) culturing can improve the in vivo‐relevance of preclinical models, while generating 3D cultures though novel bioprinting technologies can offer increased scalability and replicability. As such, there is a need to develop platforms that combine iPSC‐derived cells with 3D bioprinting to produce scalable, tunable, and biomimetic cultures for preclinical drug discovery applications. We report a biocompatible poly(ethylene glycol)‐based matrix which incorporates Arg‐Gly‐Asp and Tyr‐Ile‐Gly‐Ser‐Arg peptide motifs and full‐length collagen IV at a stiffness similar to the human brain (1.5 kPa). Using a high‐throughput commercial bioprinter we report the viable culture and morphological development of monocultured iPSC‐derived astrocytes, brain microvascular endothelial‐like cells, neural progenitors, and neurons in our novel matrix. We also show that this system supports endothelial‐like vasculogenesis and enhances neural differentiation and spontaneous activity. This platform forms a foundation for more complex, multicellular models to facilitate high‐throughput translational drug discovery for CNS disorders.

## Graphical abstract

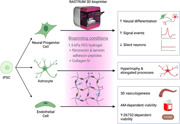

## 1 INTRODUCTION

Central nervous system (CNS) disorders are the leading cause of disability and the second leading cause of death worldwide (Feigin et al., 2017). Understanding how cells function and communicate in the CNS is critical to tackling this issue, however, the disordered human CNS consists of uniquely complex pathologies, which are prohibitively invasive to study at close proximity (Drummond & Wisniewski, 2017). For this reason, the ability of our in vivo and in vitro CNS models to adequately recapitulate a breadth of human‐specific pathologies is of utmost importance.

The majority of in vivo CNS disease studies involve rodent models, allowing the observation of cellular phenotypes in a living system where cells are in their natural multicellular, three‐dimensional (3D) environment (Dawson et al., 2018; Mcgonigle, 2014). However, significant differences in the behavior and genetics of rodent models severely impact the generalizability of these systems to human CNS diseases (McGraw et al., 2017). Contrastingly, most in vitro studies are conducted on two‐dimensional (2D) surfaces with monocultured cell lines, ignoring the 3D multicellular nature of the CNS (Birgersdotter et al., 2005). This 2D monolayer culture causes significantly altered cell morphology, function, differentiation, and gene expression compared with the in vivo environment, diminishing the physiological relevance of these cultures (Edmondson et al., 2014; Sun et al., 2006). The validity of these commonly used models for human CNS disorders is concerningly low (Liu et al., 2019; Pound & Ritskes‐Hoitinga, 2018) and as such, there is a need for further improvements to current in vitro culture systems.

The ability to create more relevant and increasingly complex in vitro models of the CNS environment has been revolutionized by the discovery of induced pluripotent stem cells (iPSCs) (Takahashi & Yamanaka, 2006). Cells differentiated from iPSCs provide a scalable solution for the production and culture of tissue‐specific cell types which closely mimic their human in vivo counterparts and allow tighter temporal control of any experimental conditions (Heikkila et al., 2009; Johnson et al., 2007; Zhang et al., 2017). iPSC‐derived cerebral organoids exhibit extensive developmental and structural aspects of the CNS (Lancaster & Knoblich, 2014), however, this model system is plagued with low reliability and lack of control over cell‐type ratios, diminishing its value for the high‐throughput screening often required during preclinical therapeutic development (Di Lullo & Kriegstein, 2017; Quadrato et al., 2017).

In addition to the benefits provided by iPSC technology, hydrogel‐based 3D cell culture represents a further biotechnological advancement in the development of biomimetic, replicable, and scalable in vitro CNS models. Others have successfully cultured neural cells in a variety of naturally derived hydrogels consisting of one or more naturally occurring proteins, such as collagen (Egawa et al., 2011) or Matrigel (a soluble mouse tumor basement membrane extract) (Choi et al., 2014), and synthetic poly(ethylene glycol) (PEG) hydrogels customized with cellular adhesion domains (Li et al., 2018). Natural hydrogels are often highly biocompatible in in vitro cell culture, however, they suffer from batch‐to‐batch variability and often have a constrained relationship between their functional groups and biomechanical properties (Aisenbrey & Murphy, 2020). Additionally, natural hydrogels often have unfavorable rheological characteristics or gelation conditions for widescale bioprinting, hence are limited in their use (Mancha Sanchez et al., 2020).

In contrast, utilizing synthetic PEG‐based hydrogels allows for highly tunable biochemical and biomechanical properties, to accurately replicate the extracellular environment being studied. This tunability takes the form of protease‐sensitive peptide sequences for biodegradation and cellular migration, covalent coupling of bioactive cellular adhesion sequences, and changes in stiffness and porosity through variations in polymer chain length and concentration (Zhu, 2010). Additionally, efficient crosslinking within PEG‐based hydrogels can be achieved through chemical/physical means without causing reductions in cellular viability (Li et al., 2018). These hydrogels are also highly compatible with high‐throughput drop‐on‐demand printing (Utama et al., 2020) which offers potential advantages in replicability and throughput over manually pipetted 3D cultures.

Relevant in vitro CNS models should support viable and controlled growth of neurons, glia and vascular cells, exhibit relevant cell‐type‐specific morphology, and facilitate appropriate physiological responses to biological or pharmacological stimuli (Hopkins et al., 2015). Challenges still remain for producing in vitro models with highly tunable scaffolds which mimic the extracellular matrix (ECM), support heterogeneous tissue architecture, and can be produced in a replicable, scalable, and high‐throughput format. Therefore, we aim to provide a customizable high‐throughput 3D bioprinting platform that can successfully support the monoculture of derived CNS neurons, glia, and vascular cells in vitro using a defined and tunable physiologically and mechanically relevant matrix, which could form the basis for future multicellular structures.

## 2 MATERIALS AND METHODS

### 2.1 Cell culture and derivations

iPSC‐derived astrocytes, brain microvascular endothelial cells (BMECs), and neurons followed methods previously published by Bardy et al. (2015), Neal et al. (2019), and Tcw et al. (2017). Specific details on cell culture and derivations can be found in the Supporting Information.

### 2.2 Immunofluorescence

For 2D samples, cells were plated on Matrigel (0.08 mg/mL) coated glass chambers. After plating (24 h), cell media was aspirated and cells were washed 3× with phosphate‐buffered saline (PBS). Cells were fixed with either 100% ice‐cold MeOH or 4% paraformaldehyde (PFA) in PBS (10 min, 22°C). For the MeOH protocol (used for 2D endothelial samples), samples were then rehydrated for 20 min in PBS (10 min, 22°C). For PFA, samples were permeabilized with 0.1% Triton‐X‐100 (10 min, 22°C). Blocking was done in 5% (v/v) fetal bovine serum in PBS (blocking solution; 1 h, 22°C). Primary antibodies were diluted in blocking solution (Table S1) and incubated overnight (4°C). The following day, cells were washed 3× with PBS and incubated with the appropriate Alexa 488/594‐conjugated secondary antibody at 1:200 in blocking solution (1 h, 22°C) (Table S2). Cells were washed 3× in PBS and mounted on a glass coverslip in 4′,6‐diamino‐2‐phenylindole dihydrochloride antifade solution (Sigma‐Aldrich). Confocal microscopy was performed using the LSM800 (Zeiss, ZEN Blue software) or the Leica SP8 (Leica, LAS X Software) and images were processed using FIJI image analysis software.

For 3D samples, cells were fixed with 4% PFA in PBS (20 min, 22°C), washed 3× in PBS (10 min, 22°C), then permeabilized with 0.1% Triton‐X‐100 (20 min, 22°C). Samples were then blocked with blocking solution (60 min, 22°C) and primary antibodies were added in blocking solution (24 h, 4°C). Samples were washed 3× (5 min [22°C], then 1 h [22°C], then 24 h [4°C]) with 0.1% v/v Tween‐20 in PBS (PBST) under gentle rocking. Samples were then incubated with secondary antibodies in blocking solution (24 h, 4°C). Samples were washed 3× in PBST as described above then incubated with 20 μΜ Hoechst 33342 (10 min, 22°C), washed 1× with PBS (22°C), and then stored in PBS at 4°C until imaged.

### 2.3 Matrix synthesis and 3D bioprinting

Three‐dimensional bioprinting was performed using the RASTRUM bioprinter from Inventia Life Sciences as described previously (Jung et al., 2022; Utama et al., 2021). Briefly, during priming and printing of the inert base layer, cells were dissociated, centrifuged, and resuspended in 200 µL of activator solution (Inventia Life Sciences) containing 3 mg/mL collagen IV at 10 × 10^6^ cells/mL for iPSC‐derived BMEC‐like cells (iBMECLs), and neural progenitor cells (NPCs) and 5 × 10^6^ cells/mL for astrocytes. Through the use of a drop‐on‐demand RASTRUM bioprinter (Inventia Life Sciences), the generation of a 3D structure was achieved by the deposition of a bioink droplet then followed by a cell‐containing droplet of activator on top, forming a covalent interaction. As the RASTRUM nozzles are optimized for generating a consistently uniform gelation on mixing of the two gel components, the physical homogeneity of the gel is far superior to that achieved by manual pipetting. Three‐dimensional structures were designed using the RASTRUM cloud (Inventia Life Sciences; Utama et al., 2020), selecting the “3D Imaging Model” or “3D Large Plug Model.” Cell‐laden matrices were printed in 96‐well plates.

### 2.4 Viability/morphology analysis

Primary human astrocytes were cultured on Matrigel‐coated (0.08 mg/well) six‐well plates in Astrocyte Growth Medium (Lonza). On the day of plating, cells were dissociated using accutase (37°C, 5 min), centrifuged (300 g, 5 min), and resuspended in activator (containing Collagen IV [Sigma‐Aldrich], Laminin 521 [BioLamina], both, or neither) at 5 × 10^6^ cells/mL. A 2 mL of the bioink (containing Arg‐Gly‐Asp [RGD], Tyr‐Ile‐Gly‐Ser‐Arg [YIGSR], both, or neither) was added into the center of a 96‐well plate to form a small droplet on the bottom of each well. An equal volume of cell‐containing activator (2 mL) was added directly into the droplet and allowed to gelate (22°C, 2 min) before adding media and left to incubate (37°C, 5% CO_2_).

To assess viability and morphology 24 h after plating the LIVE/DEAD viability/cytotoxicity kit (Invitrogen) was used. Three‐dimensional cultures were washed 1× with PBS and incubated in 2 μM calcein Astrocyte Growth Medium (AM), 4 μM ethidium homodimer‐1 (EthD‐1), and 20 μM Hoechst 33342 (Sigma‐Aldrich) (37°C, 30 min). Wells were washed 3× with PBS and fluorescent microscopy was achieved using the Cytation 3 Cell Imaging MultiMode Reader (BioTek, Gen5 v2 software), with a combination of 375, 480, and 560 nm excitation filters. Images were taken as 50 *z*‐sections spaced at 2 μm intervals on a 10× air objective, each condition was plated in duplicate and a minimum of 80 cells were imaged. Images were processed as maximum intensity projections in FIJI. For analysis, images were blinded and cell area and maximum process length were measured by manual tracing.

### 2.5 Interleukin 6 (IL‐6) enzyme‐linked immunosorbent assay (ELISA)

iPSC‐derived astrocytes were plated on Matrigel‐coated (0.08 mg/well) 96‐well plates or 3D‐printed to achieve a density of 15,000 cells/well in both conditions. After plating (24 h), media were aspirated and cells were exposed to 10 or 50 μg/mL lipopolysaccharide (LPS; *Escherichia coli* strain 0111:B4; Sigma‐Aldrich) (MilliQ H_2_O vehicle) and 50 μg/mL Toll‐like receptor 4 (TLR4) blocking antibody (14‐9917‐82; Invitrogen) in AM for 24 h. Media from above the 2D cells or above the 3D matrix were removed, centrifuged (10,000*g*, 1 min) and the supernatant stored at −80°C. IL‐6 ELISA was performed according to the manufacturer's protocols (R&D Systems product number DY206), with slight amendments, including capture antibody used at 2 μg/mL, detection antibody used at 50 ng/mL, and standard range used from 0.586 to 600 pg/mL. Absorbance at 450 nm was recorded using a BMG POLARstar Omega and analyzed using a sigmoidal dose–response (variable slope) model, with unknowns interpolated in GraphPad Prism 9.

### 2.6 Calcium imaging

After 4 weeks of differentiation, iPSC‐derived neurons were washed once with BrainPhys basal media and incubated with Fluo‐4 calcium dye (1:1000), probenecid (1:250), and PowerLoad concentrate (1:100) (Fluo‐4 Calcium Imaging Kit, Molecular Probes) in BrainPhys basal media for 30 min (37°C, 5% CO_2_) then 15 min (retention time). Fluo‐4‐containing media were removed, cells washed 1×, and left in BrainPhys basal media. The cells were then immediately imaged for 10 min at a rate of 1 frame every 0.6 s using the LSM800 under a 10× air objective and live‐cell conditions (37°C, 5% CO_2_). The analysis of calcium images was done in MATLAB (R2021b) using the “EZCalcium” toolbox developed and validated by Cantu et al. (2020), which automates the region of interest selection and generates calcium traces for each neuron body identified. These calcium traces were then quantified using the “PeakCaller” MATLAB script developed by Artimovich et al. (2017).

## 3 RESULTS

### 3.1 Matrix optimization

To identify a suitable CNS‐mimetic matrix composition, we utilized PEG‐based matrices with stiffnesses similar to that of the human cortex (1.1 and 1.5 kPa) (Weickenmeier et al., 2016) and screened a small selection of functional adhesion peptide motifs and proteins. Although ultimately we are seeking to develop a matrix suitable for bioprinting, bioprinting can impact the viability of cells due to the shear stress cells are exposed to (Shi et al., 2018). Given this, to assess the effect of matrix composition on astrocyte viability and morphology in the absence of bioprinting‐related stressors, we examined the impact of manually encapsulating (pipetting) primary human fetal astrocytes in a range of PEG‐based matrices containing various combinations of adhesion molecules/peptides. We incorporated adhesion peptide motifs found within fibronectin (RGD sequence) and laminin (YIGSR sequence) as well as the full‐length collagen IV and laminin proteins at concentrations consistent with previous literature (Antoine et al., 2014; Pereira et al., 2018; Swindle‐Reilly et al., 2012).

**Figure 1:**
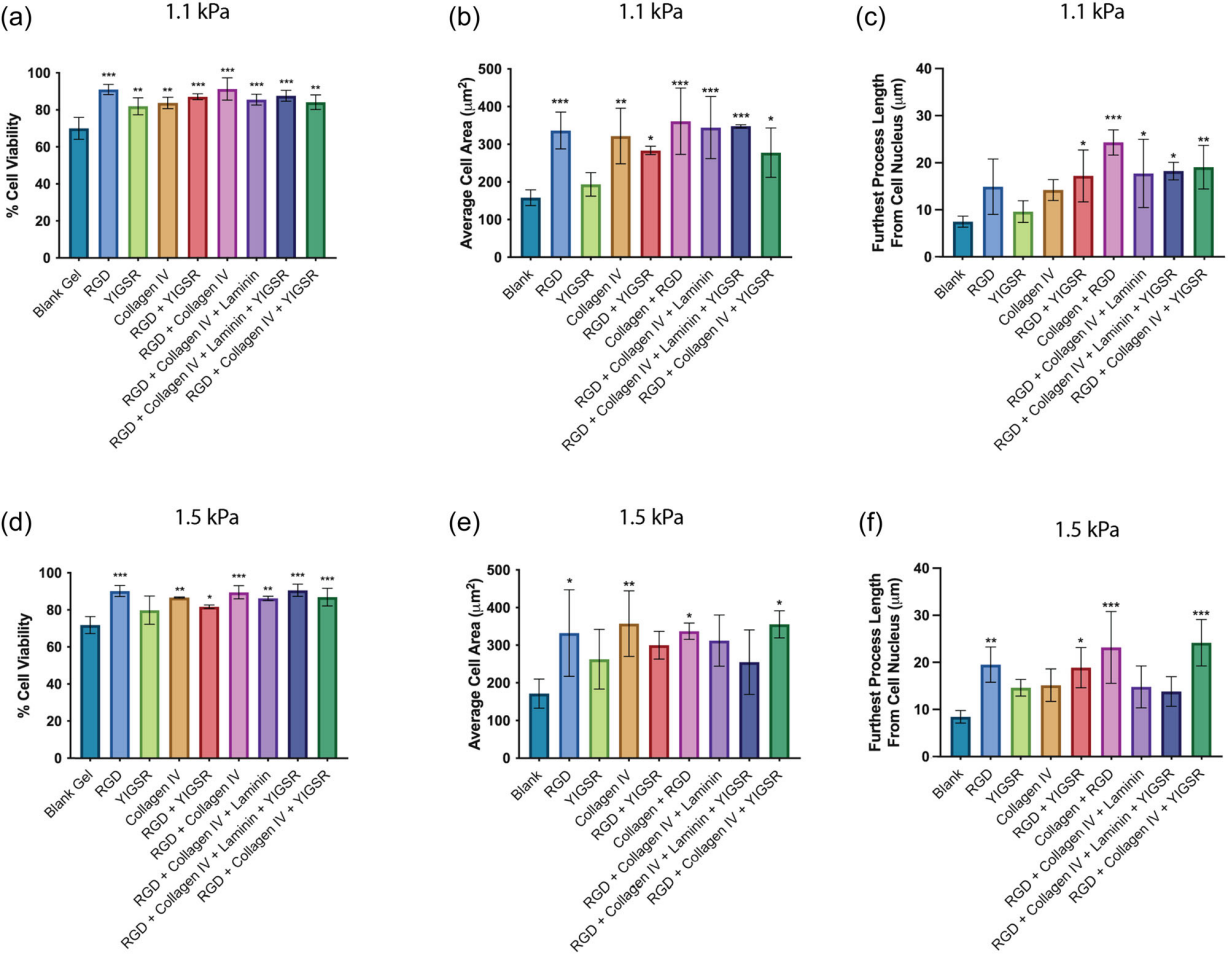
The viability, average cell area, and average longest cellular process of primary human fetal astrocytes 1‐day postencapsulation in a 1.1‐kPa PEG‐based matrix (a–c) and 1.5‐kPa PEG‐based matrix (d–f) containing various adhesion peptides and proteins (RGD, YIGSR, Collagen IV, and laminin). Data show the mean ± SD of *n* ≥ 3 independent experiments. A one‐way ANOVA with Dunnet's multiple comparison tests was used to determine whether there were statistically significant differences between each peptide/protein‐containing matrix and the blank matrix. *p < 0.05, **p < 0.01, and ***p < 0.001. ANOVA, analysis of variance; PEG, poly(ethylene glycol); RGD, Arg‐Gly‐Asp; YIGSR, Tyr‐Ile‐Gly‐Ser‐Arg.

In the 1.1‐kPa matrix, we found all of the functionalized matrix conditions to significantly increase the viability of primary human astrocytes compared with the unfunctionalized blank matrix (Figure 1a). We found similarly significant results within the 1.5‐kPa matrix, with the exception of YIGSR where it showed no significant increase over the blank matrix (Figure 1d). We then examined the extent to which astrocytes adhered to various matrix conditions by quantifying the average cell size and the maximum distance in which each cell was extending into the matrix. In contrast to cell viability, we found the different matrix compositions in both the 1.1‐kPa (Figure 1b,c) and 1.5‐kPa (Figure 1d,e) matrices to have a much more varied effect on astrocyte adherence. Incorporation of RGD and collagen IV both alone and in combination caused significant increases to cell area compared with the blank matrix regardless of stiffness (Figure 1b,e). Unique to the 1.5‐kPa matrix, the addition of laminin to the combination of RGD + collagen IV as well as the combination of RGD + collagen IV + YIGSR resulted in no significant increase in cell extension compared with the blank matrix (Figure 1e,f).

**Figure 2:**
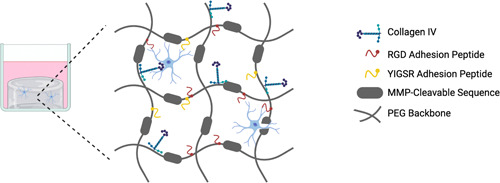
Simplified schematic of the matrix composition showing the functionalization of the matrix backbone with collagen IV, Arg‐Gly‐Asp (RGD) peptide sequence, and Tyr‐Ile‐Gly‐Ser‐Arg (YIGSR) peptide sequence. MMP, matrix metalloproteinase; PEG, poly(ethylene glycol).

Despite the matrix optimization being based upon primary human astrocyte biocompatibility, the lead matrix will ultimately facilitate a 3D culture system that aims to support multiple CNS cell types. RGD and collagen IV‐functionalized matrices provided the highest level of biocompatibility for the primary astrocytes, however despite showing no benefit to astrocyte size or process extension, YIGSR benefits the growth of other CNS cell types, such as neurons and BMECs (Grant et al., 1989; Jain & Roy, 2020). Selecting a lead matrix composition which incorporates the widest variety of motifs available, namely, RGD, YIGSR, and full‐length collagen IV provides a better opportunity for other CNS cell types to be compatible with the matrix. As such, given that the RGD + collagen IV and RGD + collagen IV + YIGSR conditions performed equally as well in the 1.5‐kPa matrix and exhibited slightly higher cell adhesion properties compared with the 1.1‐kPa matrix, we chose the 1.5‐kPa RGD + collagen IV + YIGSR composition as our lead matrix to use for further applications. A simple schematic of this matrix composition is found in Figure 2. Representative images of the changes in astrocyte morphology in the lead matrix are shown in Figure S1.

### 3.2 Bioprinted iPSC‐derived astrocytes remain viable and respond differently to inflammatory stimuli

**Figure 3:**
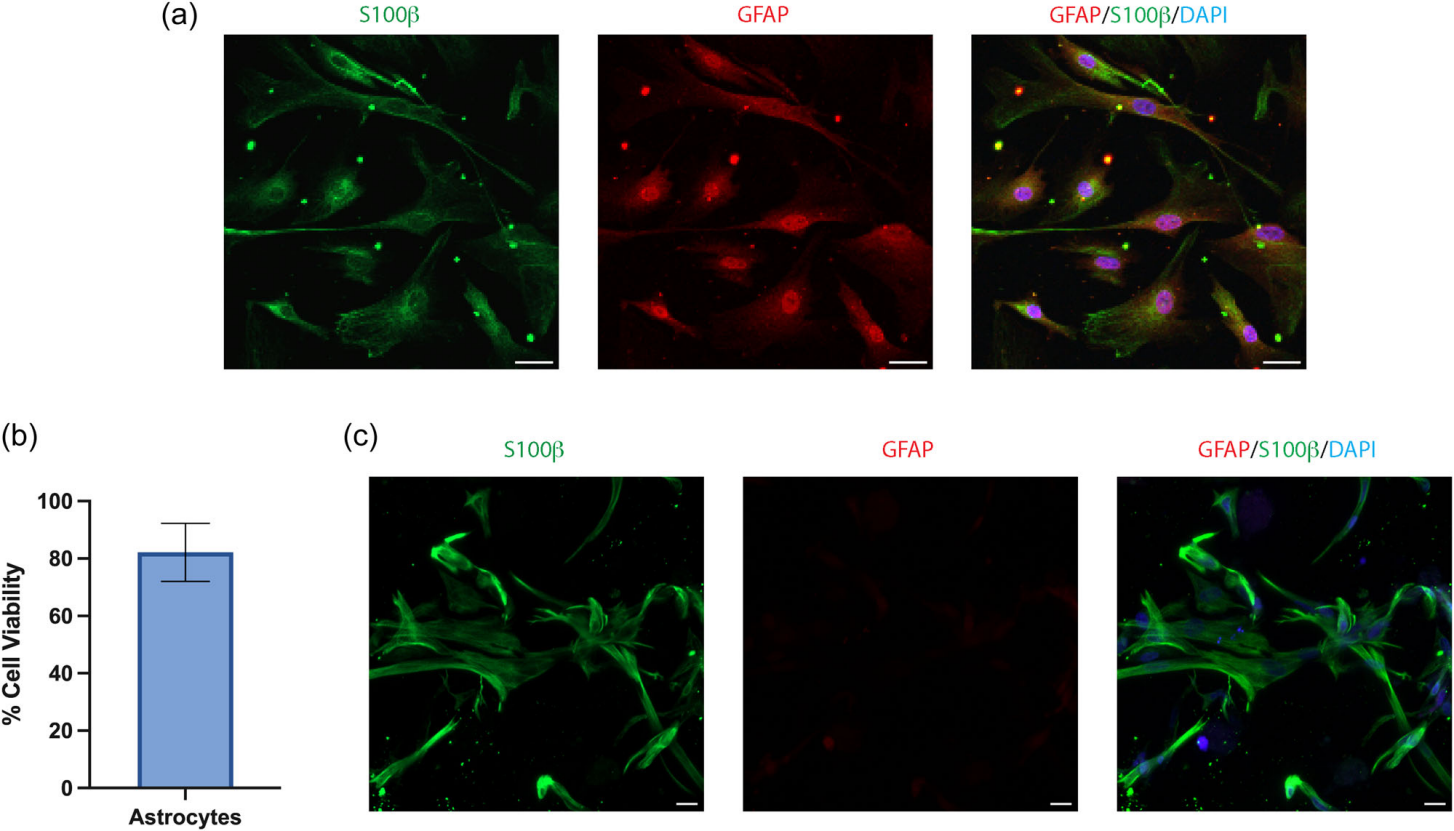
(a) Immunocytochemistry of iPSC‐derived astrocytes in 2D culture and stained for the astrocyte markers S100β (green), GFAP (red), and nuclei counterstained with DAPI (blue). (b) Viability of iPSC‐derived astrocytes 1‐day post‐bioprinting, data show the mean ± SD of *n* = 3 independent experiments. (c) Immunocytochemistry of iPSC‐derived astrocytes bioprinted in the 3D matrix and shows a maximum intensity projection of an 80 μm *z*‐stack. Scale bar = 20 μm. DAPI, 4′,6‐diamino‐2‐phenylindole dihydrochloride; GFAP, glial fibrillary acidic protein; iPSC, induced pluripotent stem cell.

Utilizing iPSC‐derived CNS cells in conjunction with 3D matrices and bioprinting technologies provides an opportunity for the production of tissue‐specific cell types within increasingly complex in vitro models of the CNS. We report the successful differentiation of iPSC‐derived astrocytes following a previously published protocol (Tcw et al., 2017) which express the astrocytic markers S100β and glial fibrillary acidic protein (GFAP) (Figure 3a). We then 3D‐printed these iPSC‐derived astrocytes with the 1.5‐kPa RGD + collagen IV + YIGSR matrix using a commercial 3D bioprinter. iPSC‐derived astrocytes were printed with good viability (82.2% ± 10.1 SD) and exhibited extensive hypertrophy of cell bodies and stretched processes characteristic of astrocyte morphology 1‐day after printing (Figure 3b,c).

**Figure 4:**
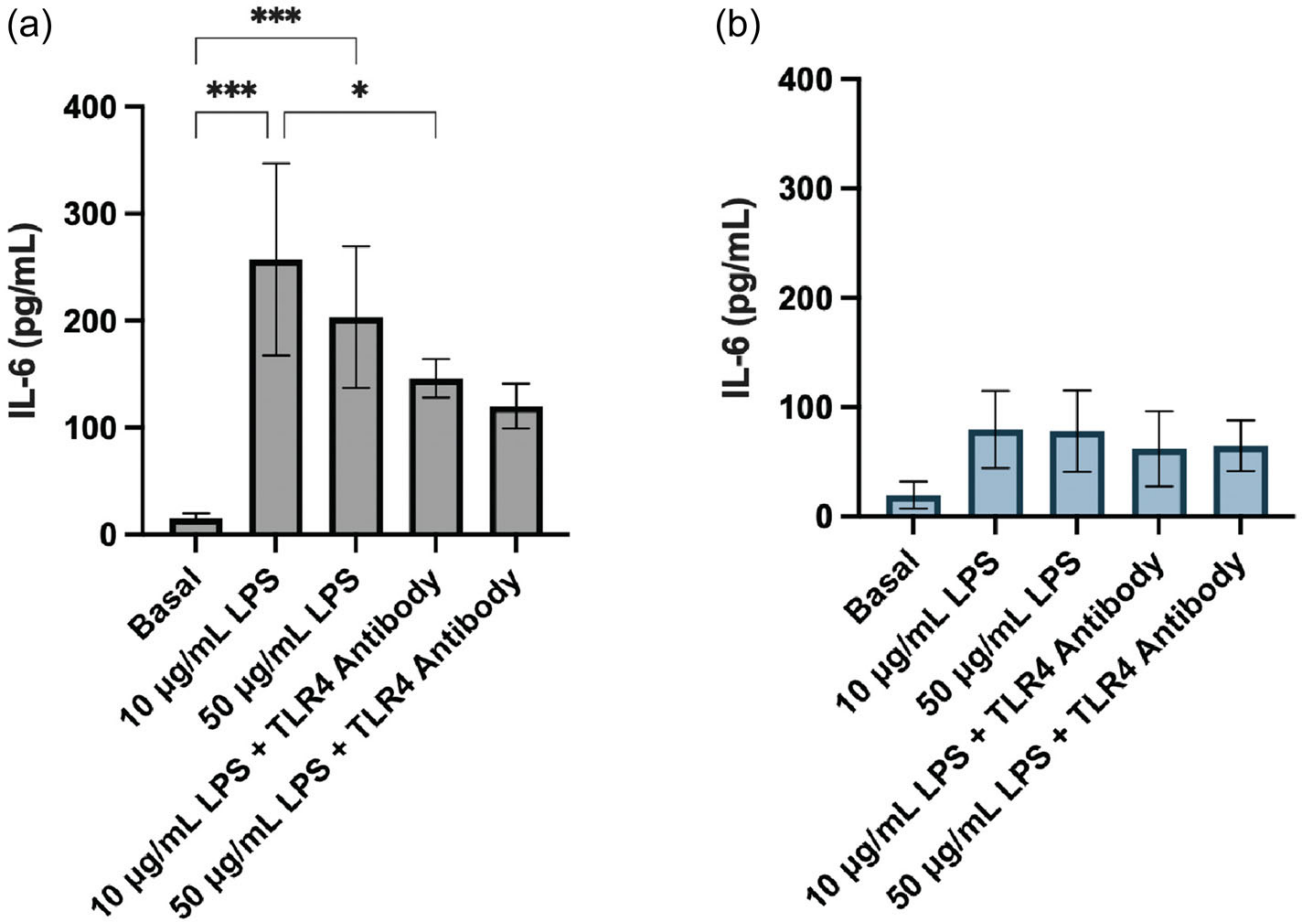
Concentration of IL‐6 secreted from (a) 2D and (b) 3D bioprinted iPSC‐derived astrocytes basally and after exposure to 10 or 50 μg/mL LPS and 50 μg/mL TLR4 blocking antibody for 24 h. Data show the mean ± SD of *n* ≥ 3 independent experiments. A one‐way ANOVA with Tukey's multiple comparison test was used to determine significant differences between conditions, relevant comparisons where a significant difference was found are shown. *p < 0.05 and ***p < 0.001. ANOVA, analysis of variance; IL‐6, interleukin 6; iPSC, induced pluripotent stem cell; LPS, lipopolysaccharide; TLR4, Toll‐like receptor 4.

Successful 3D in vitro models of the CNS should be constructed from an immunologically inert matrix scaffold and facilitate neuroinflammatory responses, of which astrocytes play a significant role (Giovannoni & Quintana, 2020). We aimed to characterize the biological response to an inflammatory stimulus and pharmacological intervention in 2D cultured and 3D bioprinted iPSC‐derived astrocytes. LPS is a bacterial‐derived polysaccharide which induces proinflammatory cytokine release through the activation of the TLR4 receptor (Gorina et al., 2011). Upon both 10 and 50 μg/mL LPS stimulation, astrocytes in 2D significantly increased their IL‐6 secretion from basal and the addition of a TLR4 blocking antibody significantly reduced the response from these astrocytes stimulated with 10 μg/mL LPS (Figure 4a). Conversely, 3D bioprinted astrocytes showed no significant increase in IL‐6 secretion upon LPS stimulation and showed no change in response to the TLR4 blocking antibody (Figure 4b).

### 3.3 Bioprinted iBMECLs remain viable and undergo vasculogenesis

**Figure 5:**
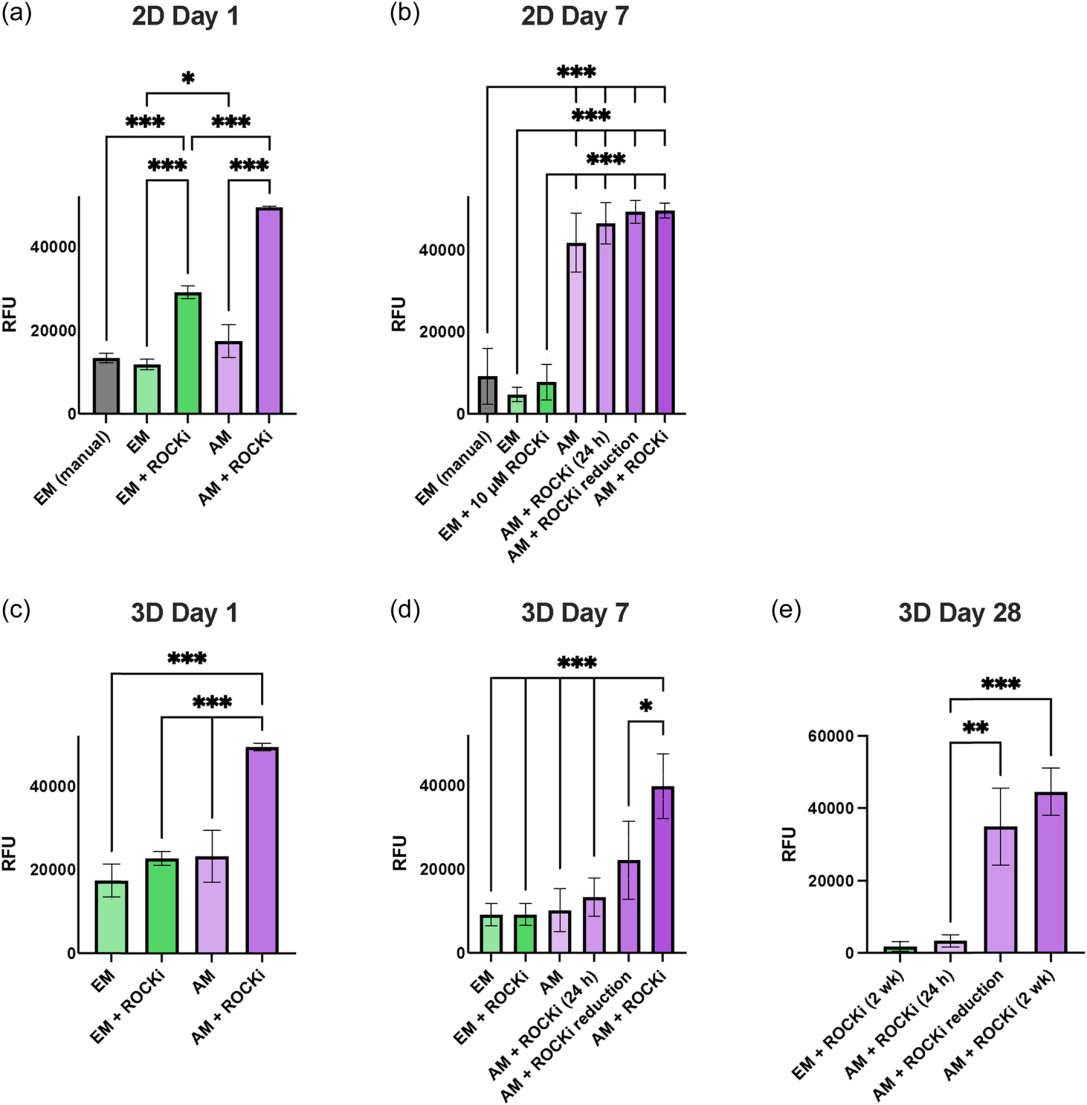
Viability of human iPSC‐derived brain microvascular endothelial‐like cells (iBMECLs) bioprinted in 2D or 3D matrices under different media conditions. Comparison of raw fluorescence units (RFU) after 4 h incubation with Cell Titer Blue in 2D cultures at day 1 (a) and day 7 (b), and in 3D cultures at day 1 (c), day 7 (d), and day 28 (e). Graphs are presented as mean ± SD of *n* ≥ 3 independent experiments. (a)–(e) are compared using a one‐way ANOVA with Tukey's multiple comparisons tests. *p < 0.05, **p < 0.01, and ***p < 0.001. AM, Astrocyte Medium; ANOVA, analysis of variance; EM, endothelial medium; iPSC, induced pluripotent stem cell; ROCKi, rho kinase inhibitor.

iBMECLs were validated by immunofluorescence for the expression of common endothelial markers (Figure S2A). After derivation, iBMECLs were then bioprinted or seeded onto 2D fibronectin/collagen IV‐coated wells. One day after printing in 2D, iBMECL viability was measured using a Cell Titer Blue assay (CTB). When cultured in the suggested media from Neal et al. (2019) (human endothelial serum‐free medium with 0.5% B27 supplement, herein referred to as endothelial medium [EM]), 2D bioprinted iBMECLs showed similar viability to nonprinted iBMECLs plated manually with a pipette (Figure 5a). However, culturing in ScienCell AM increased the viability of 2D bioprinted iBMECLs compared with EM at day 1. Additionally, by applying 10 μM of the rho kinase (ROCK) inhibitor Y‐27632 (ROCKi), cell viability of 2D bioprinted cells was increased in both EM and AM conditions, with AM + ROCKi supporting the highest viability cultures (Figure 5a). After 7 days, good viability was evidenced in all AM‐containing conditions, which reached 100% confluency, while EM conditions showed markedly lower viability (Figure 5b).

iBMECLs printed in 3D PEG matrices in comparison to 2D, displayed cell viability that was more media‐dependent. At day 1, CTB results demonstrated that AM + ROCKi provided significantly higher cell viability than EM and AM without ROCKi (Figure 5c). iBMECLs cultured in AM + ROCKi displayed a sufficient percentage of viable cells (67.71% ± 11.28 SD). iBMECLs in EM + ROCKi showed similar viability (56.75% ± 14.67 SD) but contained a high proportion of atypical nuclear staining that was delocalized from either calcein AM (live) or EthD (dead) staining (Figure S2B–D). This mirrors CTB results that indicate EM provides suboptimal conditions for iBMECL survival in this system. In the two conditions that did not contain ROCKi, calcein AM staining was rarely associated with a typical live‐cell morphology or appropriate nuclear staining (Figure S2C). Because of this, live‐cell counts were impractical, but this atypical live/dead finding corroborates CTB results suggesting poor viability in these conditions.

At day 7, 3D bioprinted iBMECLs cultured in AM + ROCKi retained high viability measured by CTB, while those cultured in EM (with or without ROCKi), and AM without ROCKi retained their comparably low viabilities (Figure 5d). Typically, ROCKi is removed after 24 h in culture (AM + 24 h ROCKi), however iBMECL viability significantly declined after the removal of ROCKi 24 h post‐bioprinting, compared with culturing in AM with daily ROCKi replacements, which had significantly higher viability than all other conditions. Unfortunately, ROCKi application may have deleterious effects on endothelial cell pathways (Cao et al., 2017). Therefore, a reduction scheme was devised to limit exposure, whereby ROCKi concentration was reduced by 2 μM per day starting on day 1 (AM + ROCKi reduction). At day 7, AM + ROCKi reduction was not significantly different from AM + ROCKi (24 h). By day 28, however, iBMECLs cultured in AM with 2 weeks ROCKi, and the ROCKi reduction scheme show comparably high viability, compared with the significantly poorer viability in EM + ROCKi and AM + ROCKi (24 h) conditions (Figure 5e). This is corroborated by live‐cell counts, showing similar live‐cell percentages for AM + ROCKi (66.58% ± 23.67 SD) and AM + ROCKi reduction (75.94% ± 3.68 SD) (Figure S2E).

**Figure 6:**
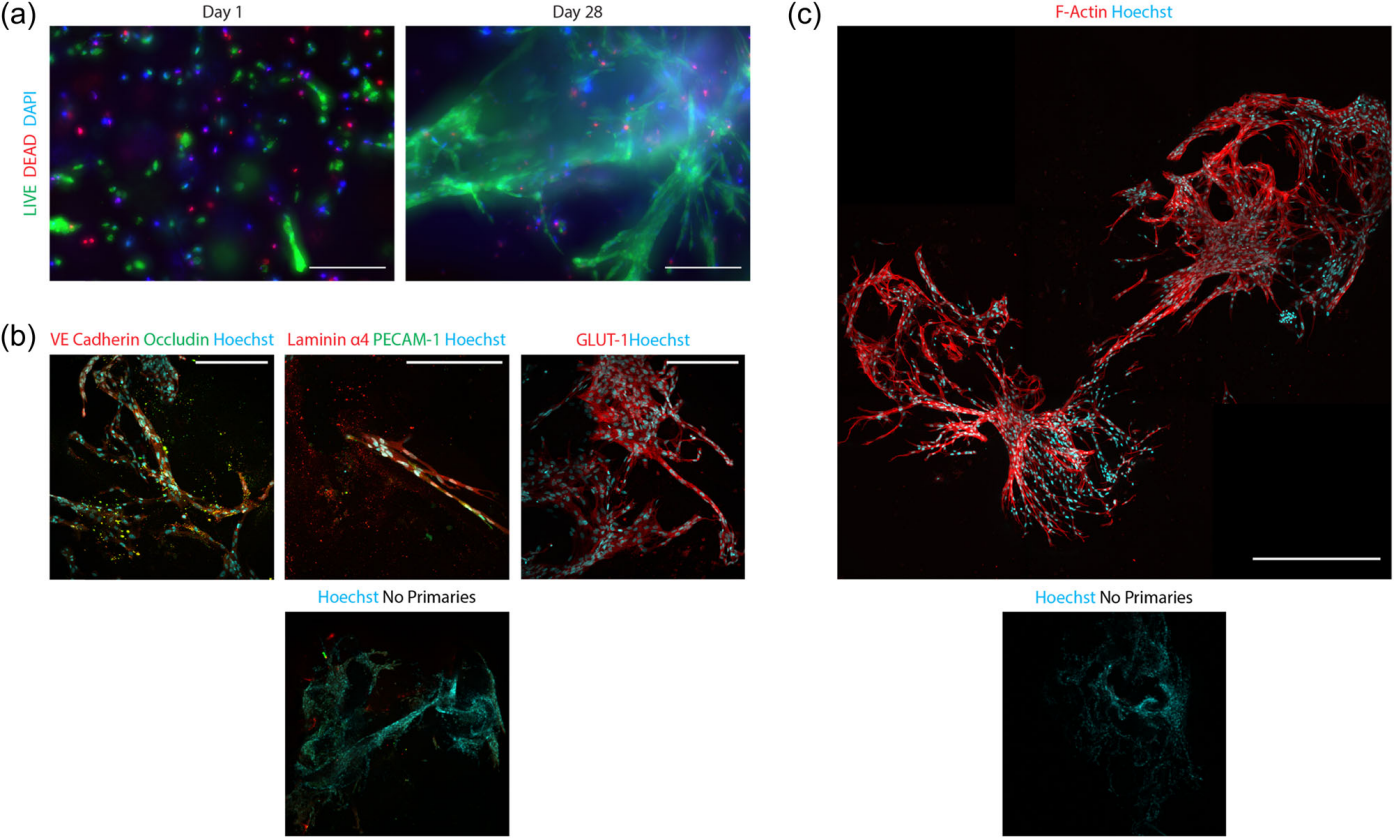
Immunofluorescence images of 3D bioprinted iBMECLs on day 28. (a) Maximum intensity projections of iBMECLs at days 1 and 28, stained with calcein AM (live) and EthD (dead). Images were taken using a 10× objective. Scale bar = 200 μm. (b) Maximum intensity projections of the expression of VE cadherin, occludin, laminin α4, PECAM‐1, and GLUT‐1 by iBMECLs. Images were taken using a 25× water objective. Scale bar = 200 μm. (c) Maximum intensity projection of F‐actin staining of iBMECLs shows the development of a complex network throughout the matrix. Image taken using a 40× dry objective. Scale bar = 500 μm. AM, Astrocyte Medium; EthD, ethidium homodimer; GLUT‐1, glucose transporter‐1; iBMECLs, iPSC‐derived BMEC‐like cells; iPSC, induced pluripotent stem cell; PECAM‐1, platelet endothelial cell adhesion molecule‐1; VE, vascular endothelial.

At day 28, these cultures expressed markers and displayed cell morphology similar to in vivo vascular formations, progressing from small, rounded, singularized cells at day 1, to elongated multicellular structures that mimic in vivo vascular networks at day 28 (Figure 6a). These iBMECL networks were stained for the adherens junctions protein vascular endothelial (VE) cadherin, the adhesion protein, platelet endothelial cell adhesion molecule‐1 (PECAM‐1), the tight junction protein, occludin, the ECM protein, laminin α4, and the functional glucose transporter‐1 (GLUT‐1) (Figure 6b). The structural complexity of the vascular networks is highlighted by the staining of F‐actin (Figure 6c).

### 3.4 Bioprinted NPCs remain viable and enhance neuronal differentiation

**Figure 7:**
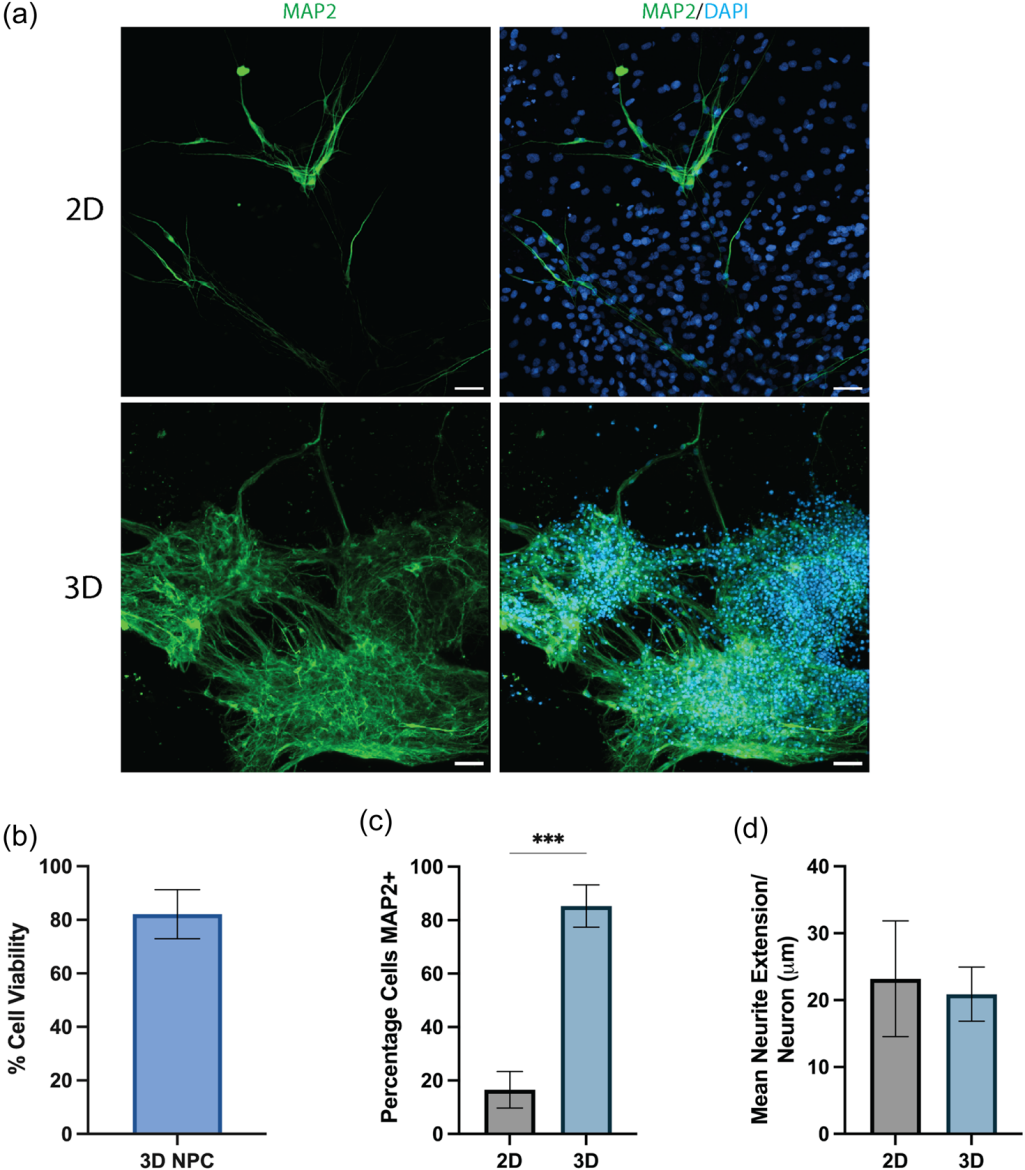
(a) Immunofluorescence images of iPSC‐derived neurons cultured in 2D and 3D bioprinted cultures after 4 weeks of differentiation. The cells were stained for the mature neuronal marker MAP2 (green) and all nuclei were counterstained with DAPI (blue). Three‐dimensional culture images are represented as a maximum intensity projection of a 100 μm *z*‐stack. Scale bar = 50 μm. (b) Viability of NPCs 1‐day post‐bioprinting. (c) Percentage of iPSC‐derived neurons positive for MAP2 and (d) average neurite extension per neuron after differentiation for 4 weeks in either 2D or bioprinted 3D cultures. Data show the mean ± SD of *n* = 3 independent experiments, an unpaired *t* test was used to determine significant differences between 2D and 3D conditions. ***p < 0.001. DAPI, 4′,6‐diamino‐2‐phenylindole dihydrochloride; iPSC, induced pluripotent stem cell; MAP2, mitogen‐activated protein 2; NPC, neural progenitor cell.

Neurons are the most widely studied cell type within the brain and are an essential aspect within in vitro models of the CNS. NPCs were able to be successfully bioprinted within our lead matrix with good viability 1‐day post printing (82.2% ± 9.2 SD) (Figure 7b) and were differentiated into neurons after 4 weeks (Figure 7a). Detection of the mature neuronal marker mitogen‐activated protein 2 (MAP2) was used to characterize the extent of neuronal differentiation between 2D and 3D culture conditions. We found differentiating neurons within 3D culture significantly increased in the percentage of MAP2 positive compared with 2D (Figure 7c), however, we observed no significant difference in the average neurite extension of MAP2 positive cells between 2D and 3D cultures (Figure 7d).

**Figure 8:**
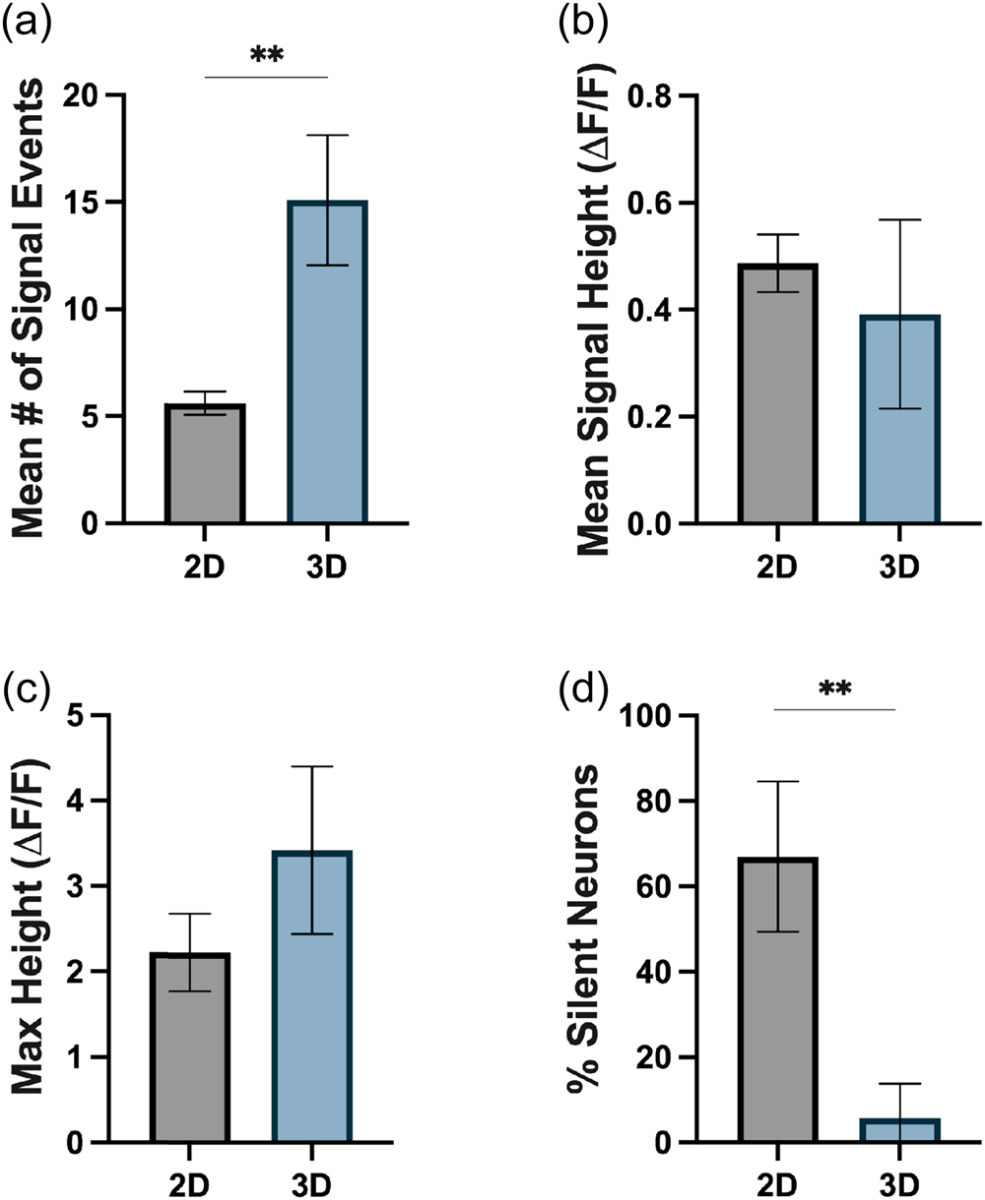
Quantification of spontaneous live‐cell calcium imaging of iPSC‐derived neurons cultured in 2D and bioprinted 3D cultures after 4 weeks of differentiation. The figure shows the quantification of (a) mean number of spontaneous calcium transients per active neuron, (b) mean signal height of each spontaneous calcium transient as measured by the change in fluorescence (Δ*F*/*F*), (c) mean maximum height of calcium transients in each neuron as measured by Δ*F*/*F*, and (d) proportion of neurons which showed no spontaneous calcium transients (termed silent neurons) over the recording period (10 min). An unpaired *t* test was used to determine significant differences between conditions. **p < 0.01. iPSC, induced pluripotent stem cell.

To generate physiologically relevant in vitro models of the CNS, 3D matrices should facilitate and support spontaneous neuronal activity. We performed a quantitative and automated analysis of live‐cell calcium imaging in both 2D and 3D bioprinted cultures of iPSC‐derived neurons following two previously published protocols (Artimovich et al., 2017; Cantu et al., 2020). In comparison to 2D culture, we found iPSC‐derived neurons cultured in 3D showed a significant increase in the number of spontaneous calcium spikes per neuron (Figure 8a) and a significant decrease in the proportion of neurons producing spontaneous calcium spikes (Figure 8d). There was no significant difference between 2D and 3D cultures in either the average signal height or the maximum signal height (Figure 8b,c). Representative calcium images and traces of iPSC‐derived neurons cultured in 2D and 3D are shown in Figure S3.

## 4 DISCUSSION

Developing physiologically relevant in vitro models of the CNS is crucial in furthering our understanding of disease pathogenesis and facilitating the screening of potential therapeutics. The success of these models depends on the ability to support the growth and function of multiple physiologically relevant CNS cells. Here we report the optimization of a novel CNS‐mimetic 3D matrix and support a viable culture of 3D bioprinted iPSC‐derived astrocytes, iBMECLs, NPCs, and enhanced neuronal differentiation and function.

During matrix optimization, we showed that regardless of matrix stiffness, addition of RGD or collagen IV to our 3D PEG matrix provided benefits to the adhesion and process extension of primary human astrocytes. The benefit of RGD and collagen incorporation within 3D matrices is consistent with a number of previous studies culturing CNS cells (Mauri et al., 2018; Scott et al., 2010). Despite concerns surrounding reductions in cell viability due to shear stressors during bioprinting (Shi et al., 2018), we were able to successfully bioprint iPSC‐derived astrocytes, NPCs, and iBMECLs within our lead matrix with a good level of viability similar to what we found using manual gelation. Recent work using extrusion‐based bioprinting of rodent astrocytes within composite gelatin‐methacryloyl matrices and NPCs within collagen‐based matrices have reported viability ranging from 75%−90%, similar to our results (de Melo et al., 2021; Ouyang et al., 2020; Salaris et al., 2019; Sharma et al., 2020). Furthermore, we show that iBMECLs bioprinted in our lead matrix, cultured in AM with tapered ROCK inhibition, remain viable up to 4 weeks. While ROCK inhibition is used in vitro for the differentiation, expansion, and protection of brain endothelial cells (Joo et al., 2012; Niego et al., 2017), it has been suggested to affect VE cadherin junctions and actin/myosin dynamics (Cao et al., 2017). After 28 days, iBMECLs cultured under the exposure‐limiting ROCKi reduction scheme showed comparable viability, marker expression, and morphology to iBMECLs cultured with 2 weeks of ROCKi. Maintained expression of junctional proteins (PECAM‐1, VE cadherin, and occludin) and functional proteins (the ECM protein laminin α4, and the glucose transporter GLUT‐1) after 28 days suggest continued iBMECL‐identity and function throughout the longitudinal culture.

In comparison to iPSC‐derived astrocytes cultured in 2D, there was a significantly lower production of IL‐6 upon LPS stimulation. Previous literature culturing primary and stem‐cell‐derived astrocytes have shown that matrix and ECM composition greatly effects the level of astrocytic activation, GFAP expression (a widely used marker for astrocyte activation) and response to various stimuli (Escartin et al., 2019; Johnson et al., 2007). Human stem cell‐derived astrocytes cultured within collagen‐based and hyaluronic acid matrices showed reductions in GFAP expression in comparison to 2D culture (Galarza et al., 2020; Placone et al., 2015; Seidlits et al., 2019). It is possible that the less exaggerated proinflammatory response to LPS observed within our 3D bioprinted iPSC‐derived astrocytes is reflective of a lower level of astrocyte activation. TLR4 expression has been shown to change between activated astrocytes and may contribute to the reduction of LPS‐stimulated IL‐6 secretion in 3D astrocytes and lack of response to the TLR4 antagonist (Wu et al., 2012).

Many current 3D endothelial models are based on manual seeding, or techniques which predefine the initial cellular architecture (e.g., sacrificial networks or subtractive fabrication), ignoring the importance of morphogenic self‐assembly for in vivo‐relevant vascular modeling (Brassard & Lutolf, 2019; Grebenyuk & Ranga, 2019). Extensive vasculogenic‐like self‐assembly displayed in our 3D bioprinted iBMECLs is reminiscent of iBMECL cultures manually seeded in Matrigel, fibrin, and GelMA matrices (Blanchard et al., 2020; Calderon et al., 2017), supporting the suitability of this platform for investigating iBMECLs function and morphology. Recent research has suggested that putative iBMECs derived using analogous methods may show a transcriptional signature characteristic of an epithelial nature (Lu, Barcia Duran, et al., 2021; Lu, Houghton, et al., 2021), potentially challenging the assumption that these cells are undergoing canonical endothelial vasculogenesis. However, it is notable that cells differentiated with an alternative (Lu, Barcia Duran, et al., 2021), a transcription factor‐mediated method, show diminished blood–brain barrier (BBB) phenotypes thus further development of differentiation protocols producing pure endothelial populations with representative BBB function is needed. One limitation of these iBMECL structures is that they cannot be perfused, hindering the examination of functions, such as barrier transport. While models incorporating fluid perfusion may be better suited to investigating questions of this nature (Kurosawa et al., 2022; Zhao et al., 2023), our model offers increased flexibility of gel composition, automaticity, and compatibility with standard plate formats. This underscores the importance of developing a suite of novel 3D techniques to improve our understanding of in vitro vascular biology.

To further validate our 3D bioprinted model for in vitro cell modeling of the CNS, we characterized the ability of our model to facilitate efficient neuronal differentiation and support neuronal function. The enhanced neuronal differentiation we observed in 3D bioprinted cultures supports previous literature using other natural and synthetic‐based matrices (Brannvall et al., 2007; Pellett et al., 2015; Ranjan et al., 2020). The enhancement of neuronal function in 3D culture is in line with previous reports of primary rodent neurons within a number of 3D matrices and found the signaling patterns to be more reflective of in vivo neuronal activity (Bosi et al., 2015; Bourke et al., 2018). Taken together, 3D bioprinting NPCs within our matrix provided a highly biocompatible environment which enhances the generation and function of iPSC‐derived neurons.

Taken together, the current study showcases the ability of our 3D bioprinted cultures to facilitate a range of biocompatibility and functionality of iPSC‐derived CNS cells. Furthermore, the tunability and scalability of this 3D bioprinted system make it an attractive platform to further develop in vitro models of CNS disorders. A key area for further development of our platform involves the coculture of multiple cell types. Coculturing neurons with various glial cells elicits cellular functions more reflective of the in vivo environment (Goshi et al., 2020). As such, coculturing iPSC‐derived neurons, astrocytes, BMECs as well as other CNS cells such as oligodendrocytes and microglia within the scalable 3D platform presented within the current study is the logical next step for developing more complex in vitro 3D models. An important consideration for these more complex multicellular models for use in preclinical drug screening is ensuring cell‐type ratios are consistent and reflective of the in vivo environment being modeled.

## AUTHOR CONTRIBUTIONS

Michael A. Sullivan and Samuel Lane contributed to the design of the work, acquisition of data, analysis of data, interpretation of data, and drafted the manuscript. Alexander Volkerling and Martin Engel contributed to the design and revised the work. Eryn L. Werry conceived the work, contributed to the design of the work, contributed to the cell line acquisition and derivations, the interpretation of data, and revised the work. Michael Kassiou contributed to the conception of the work and revised the work. All authors have read and approved the submitted version.

## CONFLICT OF INTEREST STATEMENT

Alexander Volkerling and Martin Engel are employees of Inventia Life Science Pty Ltd. Inventia has an interest in commercializing the 3D bioprinting technology.

## ETHICS STATEMENT

Approval for the use of iPSCs was gained from the University of Sydney Institutional Biosafety Committee.

## Supporting information

Supporting information.

## Data Availability

The data that support the findings of this study are available from the corresponding author upon reasonable request.
