# Supplementary material for: Three‐dimensional bioprinting of stem cell‐derived central nervous system cells enables astrocyte growth, vasculogenesis, and enhances neural differentiation/function
Source: Biotechnol Bioeng. 2023 Jul 3;120(10):3079–91. doi: 10.1002/bit.28470 (PMC10953436; doi:10.1002/bit.28470)
Supplement: Supplementary file 1 — Supporting information. [file BIT-120-3079-s001.docx]

**3D Bioprinting of Stem Cell-Derived Central Nervous System Cells Enables Astrocyte Growth, Vasculogenesis and Enhances Neural Differentiation/Function**

Michael A. Sullivan^1^, Samuel Lane^2^, Alexander Volkerling^3^, Martin Engel^3^, Eryn L. Werry^2,4*^, Michael Kassiou^2*^

^1^School of Medical Sciences, The Faculty of Medicine and Health, The University of Sydney, Sydney, Australia. ^2^School of Chemistry, The Faculty of Science, The University of Sydney, Sydney, Australia. ^3^Inventia Life Science Operations Pty Ltd, Alexandria, NSW 2015, Australia. ^4^Central Clinical School, Faculty of Medicine and Health, The University of Sydney

*Co-corresponding author: Michael Kassiou

**Email:** [michael.kassiou@sydney.edu.au](mailto:michael.kassiou@sydney.edu.au)

*Co-corresponding author: Eryn Werry

**Email:** [eryn.werry@sydney.edu.au](mailto:eryn.werry@sydney.edu.au)

**SUPPLEMENTARY INFORMATION**

**Table S1** Primary antibodies used in immunofluorescence images

| **Target** | **Host** | **Vendor** | **Product Number** | **Dilution** |
| --- | --- | --- | --- | --- |
| GFAP | rabbit | Abcam | ab7260 | 1:500 |
| S100β | mouse | Sigma-Aldrich | S2532 | 1:1000 |
| Nestin | mouse | Stem Cell | 60091 | 1:2000 |
| Pax6 | rabbit | Abcam | ab5790 | 1:50 |
| MAP2 | mouse | Invitrogen | MA5-12823 | 1:100 |
| Ve Cadherin | rabbit | Abcam | ab33168 | 1:400 |
| Occludin | mouse | Thermofisher | OC-3F10 | 1:100 |
| PECAM-1 | mouse | Abcam | ab9498 | 1:1000 |
| Laminin α4 | rabbit | Sigma-Aldrich | SAB4501719 | 1:100 |
| GLUT-1 | rabbit | Abcam | ab14309 | 1:250 |

**Table S2** Secondary antibodies used in immunofluorescence images

| **Species Reactivity** | **Host** | **Conjugate** | **Vendor** | **Product Number** | **Dilution** |
| --- | --- | --- | --- | --- | --- |
| Mouse | Donkey | Alexa Fluor 488 | ThermoFisher | A-21202 | 1:200 |
| Rabbit | Donkey | Alexa Fluor 594 | ThermoFisher | A-21207 | 1:200 |


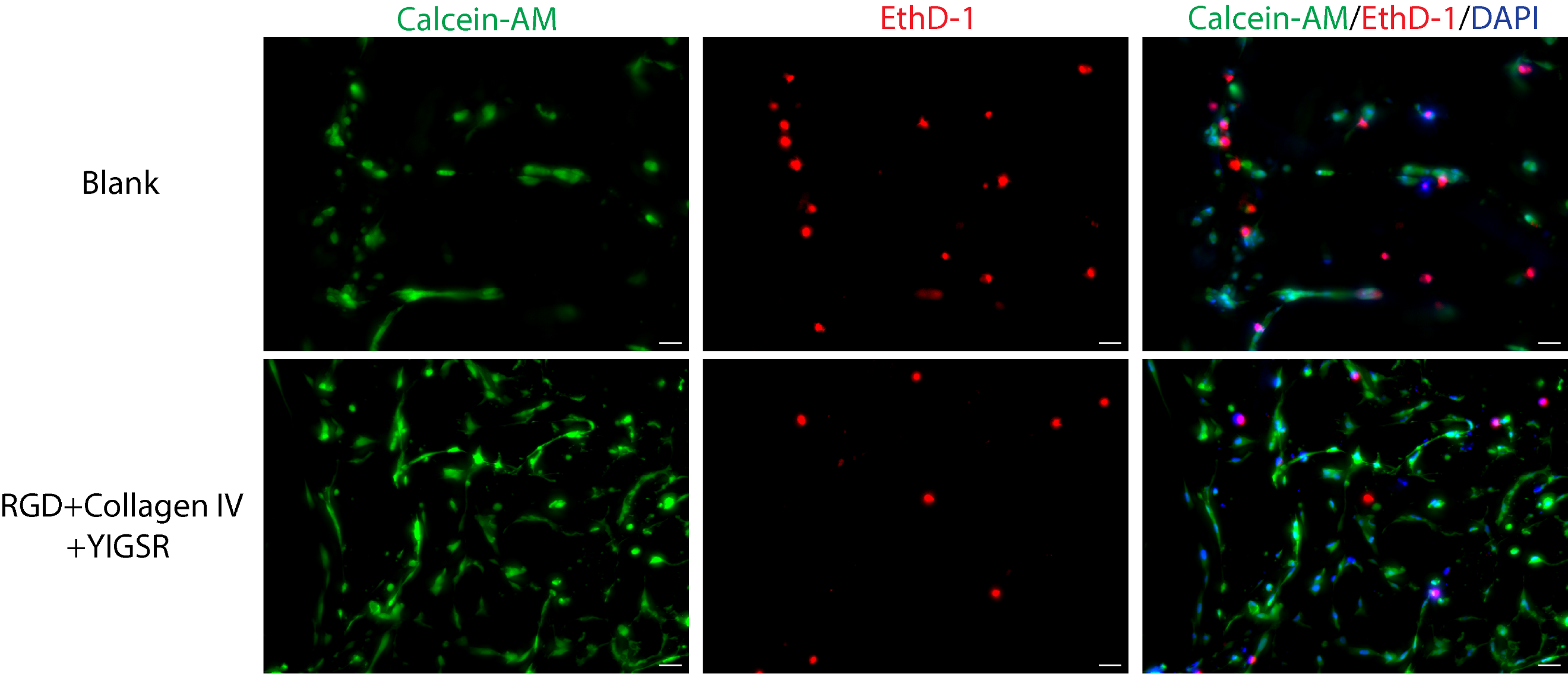


Figure S1 Representative images of primary human fetal astrocytes one day post-encapsulation in the blank unfunctionalised 1.5 kPa PEG-based matrix and lead 1.5 kPa PEG-based matrix containing RGD, YIGSR and Collagen IV. The cell membrane of live cells and the nucleus of dead cells are shown though calcein-AM and EthD-1 staining, respectively. Scale bar = 50 μm.


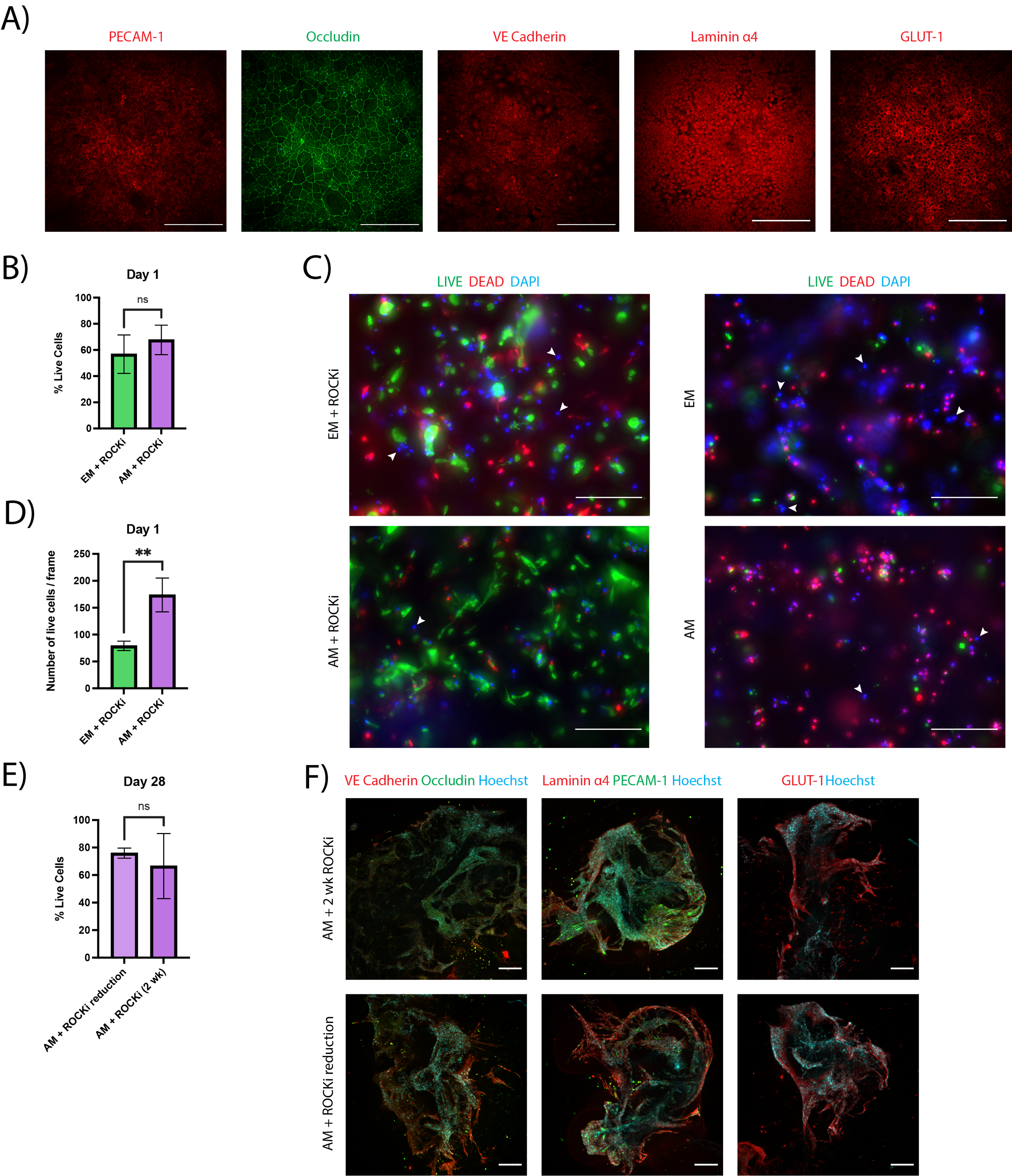


**Figure S2** A) 2D iBMECLs express VE cadherin, occludin, laminin α4, PECAM-1 and GLUT-1. Images taken using a 25× water objective. Scale bar = 200 μm. B) Percentage of live cells at Day 1 identified by Live/Dead staining. C) Maximum intensity projections of iBMECLs at Day 1 under different media conditions, stained with calcein AM and EthD. White arrows indicate examples of atypical nuclear staining. Images taken using a 10× objective. Scale bar = 200 μm. D) Number of countable live cells at Day 1 identified by Live/Dead staining. E) Percentage of live cells at Day 1 identified by Live/Dead staining. F) Maximum intensity projections of 3D iBMECLs at Day 28 under different media conditions showing expression of VE cadherin, occludin, laminin α4, PECAM-1 and GLUT-1. Images taken using a 10× dry objective. Scale bar = 200 μm. Graphs are presented as mean ± SD. B, D and E are compared using an unpaired t-test. (* p < 0.05, ** p < 0.01, *** p < 0.001).


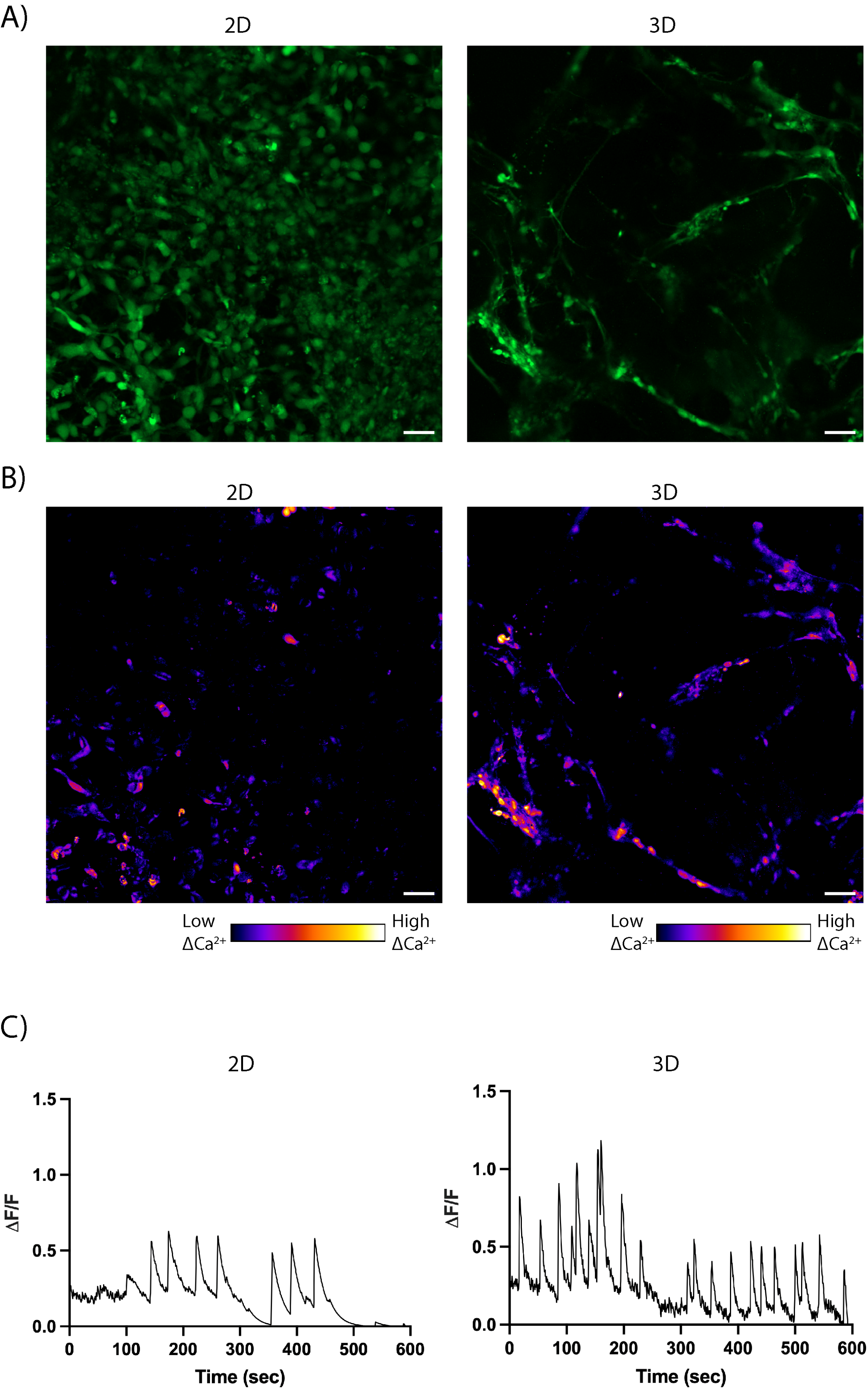


**Figure S3** Representative images of spontaneous live-cell calcium imaging of iPSC-derived neurons cultured in 2D and bioprinted 3D cultures after 4 weeks of differentiation. The figure shows A) the staining of cell membranes, B) time lapse image showing the change in calcium intensity over the 10 min recording and C) representative traces of spontaneous calcium transients from a single neuron in 2D and 3D culture.

Supplementary Methods

iPSC Line and Culture

Human control iPSCs were obtained from the Cedars-Sinai (Los Angeles, USA) cell repository. iPSCs were generated from dermal fibroblasts obtained from skin punch biopsies and reprogrammed using a non-integrating episomal plasmid and showed normal karyotyping. iPSCs were cultured in feeder free conditions on Matrigel-coated (0.08 mg/well) 6-well tissue culture plates in mTesR1 media (StemCell Technologies) supplemented with mTeSR1 5× Supplement (StemCell Technologies) at 37 ˚C and 5% CO_2_. Cultures were fed daily. For passaging of iPSC cultures, differentiated colonies were manually scratched off the bottom of the well and media was aspirated. Fresh media was added and passaged 1:6 using the StemPro EZpassage tool (Life Technologies) as per manufactures instructions.

NPC derivation

iPSCs were cultured with the addition of 10 ng/mL StemBeads FGF-2 (StemCultures). Upon reaching 80 % confluency, the media was changed to mTeSR™-E8™ supplemented with 10 ng/mL StemBeads FGF-2 and 10 μM Y-27632 (Sigma-Aldrich). After 24 h, cells were washed with PBS, dissociated with gentle cell dissociation reagent (StemCell Technologies) and incubated for 10 min (37 ˚C, 5 % CO_2_). Cells were gently pipetted up and down to ensure all cells had dislodged and formed a single cell suspension. Cells were then centrifuged (300 g, 5 min) and plated at a density of 90,000 cells/mL in STEMdiff Neural Induction Medium (StemCell Technologies) with SMADi supplement (StemCell Technologies) (NIM) and 10 μM Y-27632 on an ultra-low attachment U-bottom 96-well plate (100 μL/well) (Costar) and incubated at 37 ˚C, 5 % CO_2_. ¾ of the media was changed daily for 4 days, ensuring not to remove the embryoid bodies. Embryoid bodies were aspirated using a 200 μL wide-bore pipette tip and plated onto Matrigel-coated (0.08 mg/well) 6-well plates (10-13 embryoid bodies/well) in NIM. Media was fully replaced daily for 7 days. Cells were washed with DMEM/F12 and incubated for 70 min (37 ˚C, 5 % CO_2_) in STEMdiff Neural Rosette Selection Reagent (StemCell Technologies). Rosettes were lifted by gently dispensing DMEM/F12 onto the colonies, centrifuged (350 g, 5 min), plated onto a Matrigel-coated (0.08 mg/well) 6-well plate in NIM and incubated for 24 h (37 ˚C, 5 % CO_2_). Media was switched to neural progenitor cell (NPC) media containing DMEM/F12, 1 x N2 (Invitrogen), 1 x B27-RA (Invitrogen) and 20 ng/ml FGF2 (Abcam) and changed daily. NPCs were passaged after 4 days using accutase (Sigma-Aldrich) (5 min, 37 ˚C, 5% CO_2_) and maintained on Matrigel-coated (0.08 mg/well) 6-well plates. Further passaging was done roughly 1:3 every week.

Astrocyte derivation

NPCs were plated at 15,000 cells/cm^2^ on Matrigel-coated (0.08 mg/well) 6-well plates in NPC medium and incubated for 24 h (37 ˚C, 5 % CO_2_). Media was switched to astrocyte medium (astrocyte basal medium (ScienCell), 2 % FBS, astrocyte growth supplement and 10 U/mL penicillin/streptomycin solution) and were fed every other day. Cells were passaged with accutase (5 min, 37 ˚C, 5 % CO2) when reaching 90 % confluency, centrifuged (300 g, 5 min) and plated at the original plating density. After 30 days in astrocyte medium, astrocyte identity was then validated using IHC and used for experiments.

iBMECL Derivation
The protocol for derivation of iPSC-derived BMECs follows that outlined in Neal et al. (2019). Briefly, iPSCs were singularised with accutase, resuspended in mTeSR1 with 10 μM Y-27632 (In Vitro Technologies) and seeded at 15,800 cells/cm^2^ on 16.6 μg/cm^2^ Matrigel-coated plates. 24 h later, medium was changed to E6 medium (A1516401), and changed daily for 4 days. Medium was then changed to human endothelial serum-free media (hESFM) with 0.5 % B27 (Life Technologies), 20 ng/mL bFGF and 10 μM all-trans retinoic acid (RA; Sigma-Aldrich). After 48 h cells were then singularised and plated onto substrates coated with 400 μg/mL collagen IV (Sigma-Aldrich) and 100 μg/mL fibronectin (F1141). 24 h later, RA and bFGF were removed from the media. Assays were performed 24 h after the removal of bFGF and RA.

Neuron Derivation

Neuron derivation followed a previously published protocol outlined in (Bardy et al., 2015). For differentiation of neurons in 2D, NPCs were plated on Matrigel-coated (0.08 mg/mL) 96-well plates at 40,000 cells/well in 100 μL NPC media. The following day 50 μL of neuron media (BrainPhys media supplemented with 1:50 NeuroCult SM1 supplement, 1:100 N2 Supplement-A, 20 ng/mL BDNF, 20 ng/mL GDNF, 1 mM cyclic-AMP, 200 nM vitamin C) (StemCell Technologies) was added to the wells. A half media change was conducted every 2-3 days for 4 weeks. For 3D differentiation, NPCs were printed using the RASTRUM 3D Bioprinter at a final concentration of 5 x 10^6^ cells/mL of matrix. A full media change of NPC media was conducted every day for 3 days. 4 days post-printing, 50 μL of neuron media was added to the wells and a half media change was conducted every 2-3 days for 4 weeks.
